# The effects of the COVID-19 lockdowns on motor skill development of 6- and 7-year old children in the Netherlands: a longitudinal study

**DOI:** 10.1186/s12889-023-16733-1

**Published:** 2023-09-27

**Authors:** Anne R. den Uil, Hemke van Doorn, Mandy Schweitzer, Mirka Janssen, Ron H. J. Scholte, Vincent Busch

**Affiliations:** 1 Centre of Expertise Urban Vitality, Faculty of Sports and Nutrition, Amsterdam University of Applied Sciences, Dr. Meurerlaan 8, Amsterdam, SM 1067 The Netherlands; 2 Behavioural Science Institute, Radboud University Nijmegen, Houtlaan 4, Nijmegen, XZ 6525 The Netherlands; 3 Sarphati Amsterdam, Public Health Service (GGD), City of Amsterdam, Nieuwe Achtergracht 100, Amsterdam, WT 1018 the Netherlands

**Keywords:** COVID-19, Lockdown, Motor skill development, Motor competence, Children, Sex, Socioeconomic status, 4 skills test

## Abstract

**Background:**

The closing of schools and sports clubs during the COVID-19 lockdown raised questions about the possible impact on children’s motor skill development. Therefore, we compared motor skill development over a one-year period among four different cohorts of primary school children of which two experienced no lockdowns during the study period (control cohorts) and two cohorts experienced one or two lockdowns during the study period (lockdown cohorts).

**Methods:**

A total of 992 children from 9 primary schools in Amsterdam (the Netherlands) participated in this study (age 5 – 7; 47.5% boys, 52.5% girls). Their motor skill competence was assessed twice, first in grade 3 (T1) and thereafter in grade 4 (T2). Children in control group 1 and lockdown group 1 were assessed a third time after two years (T3). Motor skill competence was assessed using the 4-Skills Test, which includes 4 components of motor skill: jumping force (locomotion), jumping coordination (coordination), bouncing ball (object control) and standing still (stability). Mixed factorial ANOVA’s were used to analyse our data.

**Results:**

No significant differences in motor skill development over the study period between the lockdown groups and control groups (*p* > 0.05) were found, but a difference was found between the two lockdown groups: lockdown group 2 developed significantly better than lockdown group 1 (*p* = 0.008). While socioeconomic status was an effect modifier, sex and motor ability did not modify the effects of the lockdowns.

**Conclusions:**

The COVID-19 lockdowns in the Netherlands did not negatively affect motor skill development of young children in our study. Due to the complexity of the factors related to the pandemic lockdowns and the dynamic systems involved in motor skill development of children, caution must be taken with drawing general conclusions. Therefore, children’s motor skill development should be closely monitored in the upcoming years and attention should be paid to individual differences.

**Supplementary Information:**

The online version contains supplementary material available at 10.1186/s12889-023-16733-1.

## Background

The outbreak of the COVID-19 pandemic disrupted daily life across the world. For children, it drastically decreased guided physical activity possibilities through the closing of sports clubs and schools. In the Netherlands, sports and physical activities for children were fully cancelled from March 16th to April 28^th^ 2020. Until July 1st, only outside sports activities were allowed and competitions were cancelled. Throughout summer of 2020, all sports activities were possible again. Primary schools were also closed on March 16th and reopened fully on June 8th, while in many schools physical education (PE) hours were still reduced or fully dropped. Reasons for this were for example a lack of staff, prioritizing catching up on the rest of the school curriculum, and organizational challenges related to offering PE within the still existing physical distancing measures and the limited number of children allowed in class. After summer recess children went back to their regular school curriculum. In December 2020, schools were closed and sports activities were restricted again. Consequently, for several months school day routines (including two times 45 min of PE per week and at least two times 15 min of outside play per day) and physical activity habits were disrupted.

Due to the amount of time children spend in school, schools usually offer important opportunities for developing competence in motor skills. Fundamental motor skills (FMS) are the building blocks of more advanced, complex movements required to participate in sports, games or other context specific physical activity. They include object control skills (i.e. throwing and catching), locomotor skills (i.e. hopping, skipping) and balance or stability skills (i.e. one-foot balance, turning) [1]. As detailed by the developmental model of Stodden et al. [2], participation in physical activities could be an important factor influencing motor skill competence. For such competence to develop, regular participation in physical activities and deliberate, instructed practice is necessary [2–4]. While PE classes offer such opportunities and have been shown to contribute to developing motor skill competence [5], they were cancelled with the closing of schools. Several survey studies in different countries [6–12] show that during the lockdown, participation in physical activities was strongly reduced and screen time was increased, while physical fitness decreased and body mass index increased [13]. In the Netherlands, sports participation (4 times or more per month) of children between 5 and 12 years old dropped from approximately 78% in earlier months to 35% during the first lockdown [12]. Since the closing of schools and sports clubs impacted children’s opportunities for deliberate practice in their motor skills, the lockdowns were hypothesized to negatively impact children’s motor skill development.

Some initial studies on the effects of the COVID-19 lockdown on (the development of) motor skill competence indeed confirmed some effects. While two cross-sectional studies found effects in one of the measured skills [14, 15], more effects were found by Pombo et al. [16]. They found that Portuguese children aged six to nine performed significantly worse after the lockdown than before the lockdown on five out of six test items of the motor competence assessment (MCA) [17]. On the other hand, Carballo-Fazanes et al. [18] studied the long-term (4-year) changes in motor skill competence in Portuguese children that were in primary school during the COVID lockdowns and found almost no changes in motor skill competence in the total sample and in boys and girls separately, but did find effects when age and motor ability at baseline were included. Thereby, the results of studies on the effects of the COVID-19 lockdowns on motor skill competence are still mixed and inconclusive.

In addition, the study design of most studies into the possible effects of the COVID-19 lockdown(s) on the motor skill development of children thus far lacked a comparison with motor skill development during a regular schoolyear. Previously mentioned studies focused either on a cross-sectional comparisons of groups [14, 15] or on a longitudinal comparison of the COVID-19 lockdown group [16, 18], but they were not able to demonstrate altered development compared to a control group. Therefore, the goal of the present study is to compare motor skill development of Dutch children who experienced one or two lockdowns with motor skill development of children in previous years, who did not experience such lockdowns. Secondly, given the shown impact on physical activity levels [19, 20] and motor skill development [21], the interaction effects of sex, motor ability and socioeconomic status are studied.

## Methods

### Design and setting

This longitudinal study aimed to identify possible delays in motor skill development of Dutch primary school children who experienced the 2020 and 2021 pandemic lockdowns, by comparing their motor skill development to that of children before the pandemic. Nine primary schools participated that were part of the larger Dutch MAMBO project, in which the motor skills of children between 6 and 12 years old were annually assessed. The selection criteria for schools to be included in the current study were that data collection must have taken place in fall and this was done every year from 2017 to 2021 for grades 3 (age 6 years) and 4 (age 7 years), in order to minimize variability of group characteristics between cohorts. In a subsample data was also available in grade 5 (age 8 years).

#### Lockdowns

During this study two lockdowns took place:

Lockdown 1 (spring 2020) – Schools were closed for 8 weeks. Then for the next 8 weeks, physical education was still under restrictions and it was largely up to schools how to organize it. For example, some schools still fully cancelled physical education and in some schools hours were reduced. After this, there was school recess for 6 weeks.

Organized sports activities for children were fully cancelled for 6,5 weeks and for 8,5 weeks only outside sports were allowed and competitions were cancelled. Then, throughout summer, all sports activities were possible again.

Lockdown 2 (winter 2020–2021) – the Christmas holiday was started early, leading to another 8 weeks of school closure. When schools reopened, physical education was less restricted than after the first lockdown.

Organized sports activities were under restrictions since October, when competitions were cancelled once again. Per December only outside sports activities were permitted. Restrictions for sports were only fully lifted in June, resulting in sports restrictions for a total of 7,5 months.

### Participants

Nine primary schools fit the selection criteria and participated in the current study. They varied in socioeconomic status and were spread over different neighbourhoods in Amsterdam, the Netherlands. Informed consent was received for 1122 children from these schools. Of these, two children were excluded due to their age (*n* = 2). At T1, 67 children were not tested due to absence (*n* = 51), incomplete data (*n* = 2), injury/physical limitations (*n* = 12) or behaviour/lack of motivation (*n* = 2). At T2, another 61 children were not measured, due to absence (*n* = 44), incomplete data (*n* = 4), injury/physical limitations (*n* = 1), behaviour/lack of motivation (*n* = 1), change of school (*n* = 6) and withdrawn informed consent (*n* = 5). This led to a final sample of 992 grade 3 children that participated in this study (age 5 – 7, 47,5% boys). All children were assessed at least two times, in grade 3 (T1) and in grade 4 (T2). They constituted of four cohorts: control cohort 1 (CC1), control cohort 2 (CC2), lockdown cohort 1 (LC1) and lockdown cohort 2 (LC2). Two control cohorts were included to create an estimate of regular year variations compared to differences with COVID-19 years. Children in the control cohorts did not experience any lockdowns before or during the study period. Children in lockdown cohort 1 experienced the first lockdown between T1 and T2. Children in lockdown cohort 2 experienced the first lockdown before the study period and a second lockdown between T1 and T2. In control cohort 1 and lockdown cohort 1 data was also available in grade 5 (T3; seven schools). Between T2 and T3, lockdown cohort 1 thus experienced the second lockdown. Descriptive statistics on the study sample can be found in Table 1.

**Table 1:** Descriptive statistics of the study sample

Cohort	Sample size (N)	Sex (n, % boys—girls)	Age T1 (mean ± SD)	Year T1	Year T2	Year T3	N per SES group (low-medium–high)	N per motor ability group (Q1-Q2-Q3-Q4)
Control cohort 1	300	142 – 158 (47.3 – 52.7%)	6.67 ± 0.44	2017	2018	2019 (*N* = 166)	126 – 128 – 46	94 – 86 – 69 – 51
Control cohort 2	241	112 – 129 (46.5 – 53.5%)	6.55 ± 0.37	2018	2019	-	105 – 83 – 53	72 – 56 – 52 – 61
Lockdown cohort 1	213	107 – 106 (50.2 – 49.8%)	6.43 ± 0.39	2019	2020^a^	2021^b^ (*N* = 136)	94 – 80 – 39	53 – 69 – 52 – 39
Lockdown cohort 2	238	110 – 128 (46.2 – 53.8%)	6.36 ± 0.36	2020^a^	2021^b^	-	116 – 84 – 38	91 – 69 – 49 – 29

Sample sizes are based on the analyses from T1 to T2

^a^Data collection took place after the first lockdown

^b^Data collection took place after the second lockdown

SES: socioeconomic status. Q1-Q2-Q3-Q4: quartile groups

### Measures

Gross motor skill data were collected using the 4-Skills Test [22]. This test is easy to conduct in a school setting and has been found to be both reliable (ICC = 0.93, IRR -0.97) [23] and valid (*r* = 0,58) [24] for its current use. The 4-Skills Test measures 4 components of motor skill: 1. Jumping force (locomotion), 2. Bouncing ball (object control), 3. Standing still (stability) and 4. Jumping coordination (coordination). These subscales comprise 11 elements of increasing difficulty. Each element equals a ‘motor age’: the age based on demonstrated motor skill competence. This test is based on the assumption that 80% of children will achieve their respective age level. For example, 80% of 6-year-old children are expected to be able to skip. If a child successfully skips (and fails at subsequent elements), they score a motor age of 6. Comparing the total score for motor age (mean of the four components) to calendar age leads to a score for ‘motor lead’: a positive motor lead indicates that a child performs better than to be expected based on calendar age, a negative motor lead value indicates a delay compared to what is expected based on calendar age.

Socioeconomic status (SES) was determined at school-level based on the postal code of the school, leading to three SES groups (low, medium, high). Motor Ability was determined by classifying children into four groups according to their percentile score at T1 (Q1 < p25, 25 < Q2 < p50, p50 < Q3 < p75, p75 < Q4 < 100), in which percentiles scores are based on norm values determined in van Kernebeek et al. [25].

### Procedures

Data collection took place in the physical education (PE) classes during school hours. The test took approximately 45 min per group. After a general introduction, the children were divided over four PE-activities to minimize the emphasis on measuring and to prevent children from watching each other. One by one children were called to perform the test. When all children performed the test item, groups rotated to the next activity. Data was collected by a pool of test conductors. To ensure protocol compliance, all our test conductors received training on conducting the 4-Skills Test. In addition, a test leader was always present to monitor measurement quality and to coordinate the testing days. Continuity in data collection throughout the years was pursued through the presence of our research coordinator, who was responsible for measurement quality through supervision and yearly training of the test conductors.

### Data-analyses

A total score for motor age was calculated if three out of four components were tested. The ‘motor lead’ score was calculated by subtracting a child’s performance (motor age in years) from its calendar age. The median score (or 50^th^ percentile) was set at 0 by subtracting the norm median scores of the motor lead, derived from a MAMBO reference sample [25], from the raw motor lead scores of each child. This way a motor lead value of zero represents the 50^th^ percentile score (p50).

To assess whether there was a difference in motor skill development from grade 3 to 4 between the four cohorts, motor lead scores were submitted to a mixed factorial analysis of variance (ANOVA). In case of a significant interaction effect, post hoc analyses were performed using paired-sample t-tests with Bonferroni correction and a one way-ANOVA with Bonferroni-corrected multiple comparisons on the difference between T1 and T2. Two-year follow-up for control cohort 1 and lockdown cohort 1 was done in the same way, by four mixed factorial ANOVA’s with Bonferroni-adjusted post-hoc tests where needed.

Furthermore, the potential effect modification by sex, SES and motor ability was analysed. Each of these were added to the ANOVA separately, resulting in three extra mixed factorial ANOVA’s. In case of a significant three way interaction, similar follow-up analyses were performed.

Only complete cases were used. Statistical significance was set at p < 0,05 for all tests. All statistical analyses were executed using IBM SPSS Statistics for Windows, Version 28 (Armonk, NY: IBM Corp).

## Results

### General effects of time, sex, motor ability and SES in the total sample

The general effect of time was not significant (*p* = 0.467), meaning that the average motor lead of the total sample remained similar between T1 and T2. Boys showed more motor skill development in one year than girls (*p* = 0.020), while there was no difference in motor skill development over one year between SES groups (*p* = 0.434). There was a significant difference in motor skill development between all four motor ability groups (*p* < 0.001), for details see the Supplementary Information.

### Differences at T1 and T2 between cohorts

There was a significant difference in motor lead on both T1 (*p* < 0.001) and T2 (*p* = 0.005) between several cohorts, thus showing yearly variations in motor skill competence between cohorts (for details see Table 2).

**Table 2:** Motor lead (years) at T1 and T2 for the four cohorts and the results of the repeated measures ANOVA + paired samples t-tests

**Cohort**		Motor Lead T1		Motor Lead T2			
	*N*	*M *^*a*^	*SD*	*M *^*b*^	*SD*	T2 – T1	
**Control cohort 1**	300	-0.223	1.092	-0.264	1.200	-0.041	Time*cohort F(3,988) = 4.186, *p* = 0.006*SD*
**Control cohort 2**	241	0.014	1.060	0.081	1.175	0.067	
**Lockdown cohort 1**	213	-0.053	0.934	-0.160	1.195	-0.107	
**Lockdown cohort 2**	238	-0.380	0.936	-0.218	1.116	0.162*	

^a^significant differences between CC1-CC2, CC2-LC2 and LC1-LC2

^b^significant differences between CC1-CC2 and CC2-LC2

^*^significant change in motor lead from T1 to T2

### Differences in motor skill development between cohorts

**Fig. 1:**
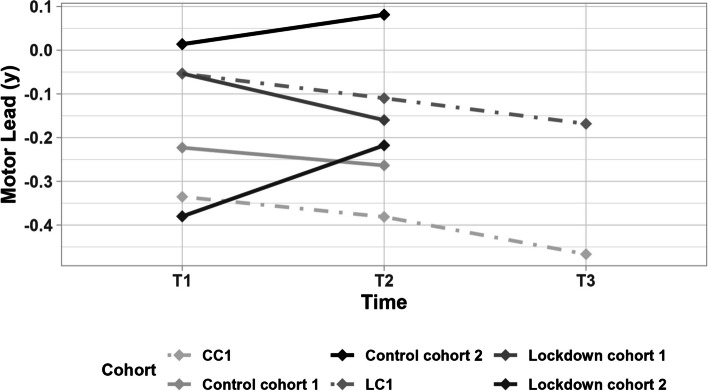
The development of motor lead in years from T1 to T2 for the four different cohorts and from T1-T2-T3 for the two follow-up cohorts (control cohort 1 (CC1), lockdown cohort 1 (LC1)). Two-year follow-up is done in only 7 schools, which accounts for the differences observed between the samples

Our results show an interaction effect of time*cohort (*p* = 0.006): there was a significant difference in motor skill development over the study period between lockdown cohort 1 and lockdown cohort 2, see Fig. 1 (*p* = 0.008). Lockdown cohort 2 shows a significant increase in motor lead between T1 and T2 (*p* = 0.003), while the other cohorts show no changes between T1 and T2 (*p* > 0.05), see Table 2. These results indicate that both lockdown cohorts show no decline in motor skill development over the study period compared to the control cohorts.

### Effects of sex, motor ability and SES

**Fig. 2:**
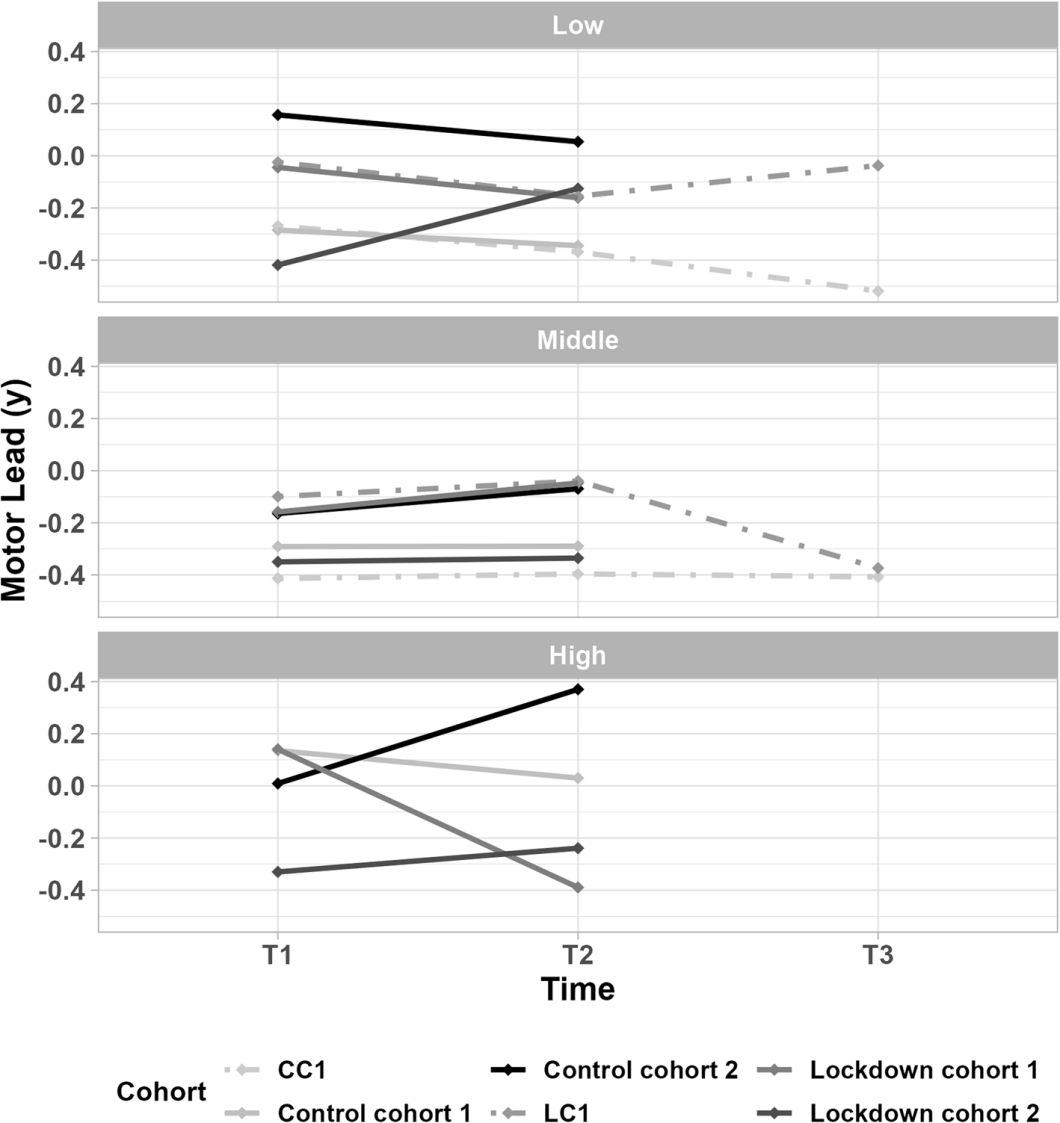
The development of motor lead in years in low-, medium- and high-SES groups from T1 to T2 for the four different cohorts and from T1-T2-T3 for the two follow-up cohorts (control cohort 1 (CC1), lockdown cohort 1 (LC1)). Two-year follow-up is done in only 7 schools, which accounts for the differences observed between the samples. Therefore, no data on the high-SES group is available in this sample

Sex and motor ability did not modify the interaction between time and cohort. Regarding SES, there was a significant three-way interaction effect (*p* < 0,001): children in the low-SES group of lockdown cohort 2 showed increased motor skills development (change in motor lead) compared to the other cohorts (see Fig. 2). Specifically, lockdown cohort 2 (low SES) showed a significant increase in motor lead between T1 and T2 (*p* < 0,001), while the other cohorts did not. For medium SES, no interaction effects were significant. In the high-SES group, children in lockdown cohort 1 developed significantly poorer than in control cohort 2 (*p* < 0.001) and lockdown cohort 2 (*p* = 0.010), but children in control cohort 2 also progressed more than children in control cohort 1 (*p* = 0.043). In this high-SES group, motor lead significantly increased from T1 to T2 in control cohort 2 (*p* < 0.001), but significantly decreased in lockdown cohort 1 (*p* = 0.002). For details, see Supplementary Information.

### Two year follow-up

Two year follow-up data show no significant difference in motor skill development between the two cohorts (*p* = 0.968), with motor lead not significantly changing over time in both cohorts (see Supplementary Information). Similar to the four-cohort analyses, there were no modification effects of sex and motor ability. A significant interaction was found for the effect of SES (*p* = 0.012). Specifically, in the low-SES group, motor lead of children in the control cohort significantly decreases with time from T1 to T3 (*p* = 0.035), while in the lockdown cohort this is not the case (*p* > 0.05). In the medium-SES group there was a difference in motor lead development between cohorts from T2 to T3 (*p* = 0.038), with children in the lockdown cohort showing a significant decrease in motor lead from T2 to T3 (*p* = 0.009) and from T1 to T3 (*p* = 0.034), while the motor lead of children in the control cohort shows no significant changes. No data on the high-SES group is available in this sample.

## Discussion

In this study we compared motor skill development of primary school children in Amsterdam (the Netherlands) over a one and two year period during the 2020 and 2021 COVID-19 lockdowns to that in pre-COVID years 2018 and 2019. Our results show that the COVID-19 lockdowns in the Netherlands did not result in significantly poorer motor skill development among the children in our study. Sex and motor ability did not cause significant interaction effects between the lockdowns and children’s motor skill development. It was anticipated that especially children in low SES areas would be most adversely affected in terms of their motor skill development. Our results, however, showed that their motor skills developed even better during the lockdown years compared to the control cohorts that were measured in the years before the lockdowns. Unexpectedly, it was the motor skill development of the high-SES group that had worsened in the lockdown cohort 1 compared to the other cohorts.

From previous research it might be expected that children affected by the Covid-19 lockdowns would show poorer motor skill development compared to children in control cohorts [14–16, 18]. However, our findings do not support this. This might be due to several factors. Firstly, there are relatively few studies done on this topic, and those that have been done show significant differences in study designs, e.g. Abe et al. [15] and Vrieswijk et al. [14] made cross-sectional comparisons between two different samples: one before the COVID-19 lockdown and one after the COVID-19 lockdown. It is possible that the detected differences in these studies are simply natural variation between samples, especially when samples are not identical in terms of for example schools or socioeconomic status. In our data we observed variation in motor skill competence at T1 between cohorts, despite matching of the samples in terms of participating schools and period of measurement. Secondly, it could also be that only young children (< 6 years) were affected in their motor skill development and that motor skill development of older children is more robust against changes in the environment, since both Vrieswijk et al. [14] and Carballo-Fazanes et al. [18] mostly found significant differences in the youngest age group and not in the older children.

An important issue in comparing studies on the effects of the lockdowns between countries is that the characteristics of the lockdowns varied widely between countries. For example, in Portugal children were home-schooled until the end of the schoolyear and all physical activities for children were cancelled until September 2020, while children were discouraged to spend a lot of time outside [16]. In Canada, for example, many cities even closed their playgrounds and parks [10], depriving children from outdoor physical activity opportunities. In the Netherlands, families were allowed to go outside and engage in outside activities freely. While school and sports groups were closed for Dutch children as well, their outside free-play activities were not tightly restricted. Additionally, perceptions of restrictive measures [26] and compliance with restrictive measures [27] differed between countries. Possibly, the lockdown in the Netherlands did not restrict physical activities to the extent that it impacted the development of motor skill competence. In other countries this may have been different.

It seems like many factors during the lockdown come into play in shifting of physical activities and it appears that we cannot simply state that COVID-19 related restrictive measures have resulted in deterioration of motor skill competence in children. Certain factors are directly related to COVID-19, such as imposed lockdown measures, perception of and compliance to these measures [26, 27], the amount of attention for maintenance of physical activity participation during the pandemic (both on a country level and on a school and parent level) and personal factors that define to what extent children were actually facilitated and able to maintain a certain level of physical activities [28, 29]. Other factors are related to motor skill development: it becomes more and more apparent that motor skill development in general is the result of complex interrelations of factors. While it is often assumed that more physical activity automatically leads to more motor skill development, many studies do not seem to support this [30]. While Stodden et al. [2] describe multiple factors (physical activity, physical fitness, perceived motor competence, weight status) interacting and leading to motor skill development, other factors beyond these, such as environmental factors, have been identified [31, 32]. Although a long-term impact of the COVID-19 lockdowns on motor skill development could come to light in the future, it could therefore also be that the direct effects of the pandemic lockdowns are, at least on a group level, smaller than expected.

General conclusions on motor skill development during the pandemic worldwide are therefore at least difficult to make. In the Netherlands, it seemed like there quickly was increased attention to maintaining physical activities. Our explorative questionnaire amongst PE teachers in Amsterdam revealed that several teachers offered digital classes or developed at home exercises during the lockdown. Similarly, especially during the first lockdown, some parents were also at home and were potentially more available to partake in activities with their children. In a Dutch survey, parents indicated that they encouraged their children to do physical activities more than usual, and they often did this by doing activities together with their child [33]. It is also possible that children played outside more than usual, since school is an important source of sedentary time [34, 35]. That same Dutch survey indicated that the percentage of children that played outside more than 10 h a week increased. It should be noted that these results are based on self-report surveys and no direct measurements of physical activity were done [33]. Nonetheless, such undertaken home activities may have prevented significant delays in motor skill development.

Our results show no influence of sex and motor ability on lockdown effects. Such modifying effects of sex could have been present due to the dis- and reappearance of typical sex differences in physical activity levels [19] found during the lockdown [9, 36]. However, this did not lead to sex-specific effects on motor skill development in our study, which is (largely) in line with the studies by Pombo et al. [16] and Carballo-Fazanes et al. [18]. An a priori hypothesis was also that missing out on PE, and possibly other school-based programs such as Motor Remedial Teaching, would negatively impact children’s motor skill development, especially among those with pre-existing motor delays. Since children with motor delays are found to be less active during free play time than children without motor delays [20], instructed practice at school is expected to be essential for their motor skill development. Although Vrieswijk et al. [14] saw indications that children in the lowest tertile indeed showed the largest decrease in motor skill competence, Carballo-Fazanes et al. [18] found that mostly children with higher motor skill competence at baseline showed lower motor skill development, while children with lower motor skill competence at baseline even improved their motor skill competence. Our study does not confirm either of these results, since we found no influence of motor ability on the presence of lockdown effects in terms of motor skill development.

Similarly, our results regarding the effects of COVID-19 lockdowns on motor skill development in different SES groups are not fully in line with what to expect from previous studies. Since children with lower SES tend to have lower motor skill development [21], these children could have been impacted more by the cancellation of instructed practice at school. However, we found that children in the low-SES group did not have altered motor skill development after the first lockdown while children in the high-SES group did. This might be because children in the low-SES group are already used to participating in unstructured activities (without trainer/teacher) such as outside play [37] and do not depend on sports groups to practice their skills. This is different for children in the high-SES group, who are used to going to sports groups [38] and specifically those were cancelled during the lockdown. Possibly, due to the temporary nature of the restrictive measures, these children more often chose to resort to less active activities and therefore showed slowed motor skill development. However, the high-SES group contains children from only one school, meaning that results of this group could be due to other factors than SES as well. Over the two-year period, we saw a decline in motor skill development in the middle-SES group, while we saw better motor skill development during the pandemic compared to the pre-pandemic cohort in the low-SES group.

Lastly, the fact that children (especially in the low-SES group) showed greater motor skill development in the schoolyear after the first covid lockdown (lockdown cohort 2) than in the years before was also unexpected, since they had experienced two lockdowns. We therefore expected these children to also show slowed motor skill development and possibly even more than children who experienced only the first lockdown. In summary, these results underline the complexity of motor skill development. Assumptions based on one factor that is expected to impact motor skill development, such as participation in physical activities, are not confirmed in this study. As described in Bronfenbrenner’s ecological systems theory [39], our study shows that a child’s development should be seen as a complex system in which many layer’s and factors interact. To have a valuable impact on children’s motor skill competence, this complex systems view should thus shape our approach.

### Strengths and limitations

The current study has a number of strengths. These include the one year longitudinal design that was extended with a two year follow-up, the matching of the study samples and the use of an experienced team for our motor skill measurements. Furthermore, the inclusion of multiple control and lockdown cohorts allowed us to study average fluctuations between cohorts. Some limitations of this study must also be pointed out. We only investigated the total motor skill score, which precluded us from finding effects in specific skills, as done in Pombo et al. [16] and Carballo Fazanes et al. [18]. Secondly, this study only included one school with high socioeconomic status, which was missing in the two year follow-up analyses. Therefore, our conclusions on high SES should be interpreted with caution. Extending on that, a larger sample size would have made our conclusions, especially on the modifiers, more reliable. Additionally, we did not include other possible modifiers, such as weight status and physical activity participation [40, 41], in our analyses. Similarly, we did not include other external factors that could play a role, such as the social and physical home environment and school and city initiatives during the pandemic. The inclusion of such modifiers and factors could have provided us with a more complete picture and thereby more direction in the interpretation of our results.

## Conclusions

In conclusion, it seems like the effects of the COVID-19-lockdowns on children’s motor skill development are not as straightforward as they may have seemed at first. Since children’s willingness to participate in physical activity has not changed [42] nor their perceived motor competence [43], the COVID-19 lockdowns may not have had permanent detrimental effects on children’s activity choices. While the short-term disruptions clearly resulted in shifts in physical activity patterns and possibilities for deliberate practice temporarily, children’s motor skill development might be more resilient to changes in the environment than we expected. However, since even within the Netherlands, both school policies and the social and physical environment of children vary so widely, generic conclusions to motor skill development during the pandemic lockdowns in the Netherlands may still not show the complete picture. Although we do not find generic decreases in motor skill development since the pandemic lockdowns, certain (groups of) children, might still have deteriorated motor skill competence, as can be seen in our analyses on SES groups. It will thus be important for parents and physical education teachers to closely monitor motor skill development in the upcoming years and to pay attention to individual differences.

### Supplementary Information

**Additional file 1:** **Appendix Figure S1.** The development of motor lead in years in boys and girls from T1 to T2 for the four different cohorts and from T1-T2-T3 for the two follow-up cohorts (control cohort 1, lockdown cohort 1). Two-year follow-up is done in only 7 schools, which accounts for the differences observed between the samples. **Appendix Figure 2.** The development of motor lead in years in the four motor ability groups (quartiles) from T1 to T2 for the four different cohorts and from T1-T2-T3 for the two follow-up cohorts (control cohort 1, lockdown cohort 1). Two-year follow-up is done in only 7 schools, which accounts for the differences observed between the samples. **Appendix Table 1.** Multiple Comparisons with Bonferroni correction between the four cohorts on the difference between motor lead on T1 and T2. **Appendix Table 2.** Analysis on the role of sex in the difference in motor lead (in years) development from T1 and T2 between the 4 cohorts. **Appendix Table 3.** Analysis on the role of SES in the difference in motor lead (in years) development from T1 and T2 between the 4 cohorts. **Appendix Table 4.** Analysis on the role of motor ability in the difference in motor lead (in years) development from T1 to T2 between the 4 cohorts. **Appendix Table 5.** Means and standard deviations of motor lead (in years) for T1, T2 and T3 in the two cohorts. **Appendix Table 6.** Analysis on the role of sex in the difference in motor lead (in years) development from T1 to T3 between the 2 cohorts. **Appendix Table 7.** Analysis on the role of motor ability in the difference in motor lead (in years) development from T1 to T3 between the 2 cohorts. **Appendix Table 8.** Analysis on the role of SES in the difference in motor lead (in years) development from T1 to T3 between the 2 cohorts.

## Data Availability

The datasets used and/or analysed during the current study are available from the corresponding author on reasonable request.

## Abbreviations

T1–T2–T3: Time point 1, 2 and 3
PE: Physical education
FMS: Fundamental motor skills
CC1: Control cohort 1
CC2: Control cohort 2
LC1: Lockdown cohort 1
LC2: Lockdown cohort 2
SES: Socioeconomic status
Q1–Q2 – Q3 – Q4: Quartile groups
ICC: Intraclass correlation
IRR: Inter-rater reliability
ANOVA: Analysis of Variance
