# Supplementary material for: The effects of the COVID-19 lockdowns on motor skill development of 6- and 7-year old children in the Netherlands: a longitudinal study
Source: BMC Public Health. 2023 Sep 27;23:1871. doi: 10.1186/s12889-023-16733-1 (PMC10523696; doi:10.1186/s12889-023-16733-1)
Supplement: Supplementary file 1 — Additional file 1: Appendix Figure S1. The development of motor lead in years in boys and girls from T1 to T2 for the four different cohorts and from T1-T2-T3 for the two follow-up cohorts (control cohort 1, lockdown cohort 1). Two-year follow-up is done in only 7 schools, which accounts for the differences observed between the samples. Appendix Figure 2. The development of motor lead in years in the four motor ability groups (quartiles) from T1 to T2 for the four different cohorts and from T1-T2-T3 for the two follow-up cohorts (control cohort 1, lockdown cohort 1). Two-year follow-up is done in only 7 schools, which accounts for the differences observed between the samples. Appendix Table 1. Multiple Comparisons with Bonferroni correction between the four cohorts on the difference between motor lead on T1 and T2. Appendix Table 2. Analysis on the role of sex in the difference in motor lead (in years) development from T1 and T2 between the 4 cohorts. Appendix Table 3. Analysis on the role of SES in the difference in motor lead (in years) development from T1 and T2 between the 4 cohorts. Appendix Table 4. Analysis on the role of motor ability in the difference in motor lead (in years) development from T1 to T2 between the 4 cohorts. Appendix Table 5. Means and standard deviations of motor lead (in years) for T1, T2 and T3 in the two cohorts. Appendix Table 6. Analysis on the role of sex in the difference in motor lead (in years) development from T1 to T3 between the 2 cohorts. Appendix Table 7. Analysis on the role of motor ability in the difference in motor lead (in years) development from T1 to T3 between the 2 cohorts. Appendix Table 8. Analysis on the role of SES in the difference in motor lead (in years) development from T1 to T3 between the 2 cohorts. [file 12889_2023_16733_MOESM1_ESM.docx]

# Supplementary Information

## Figures


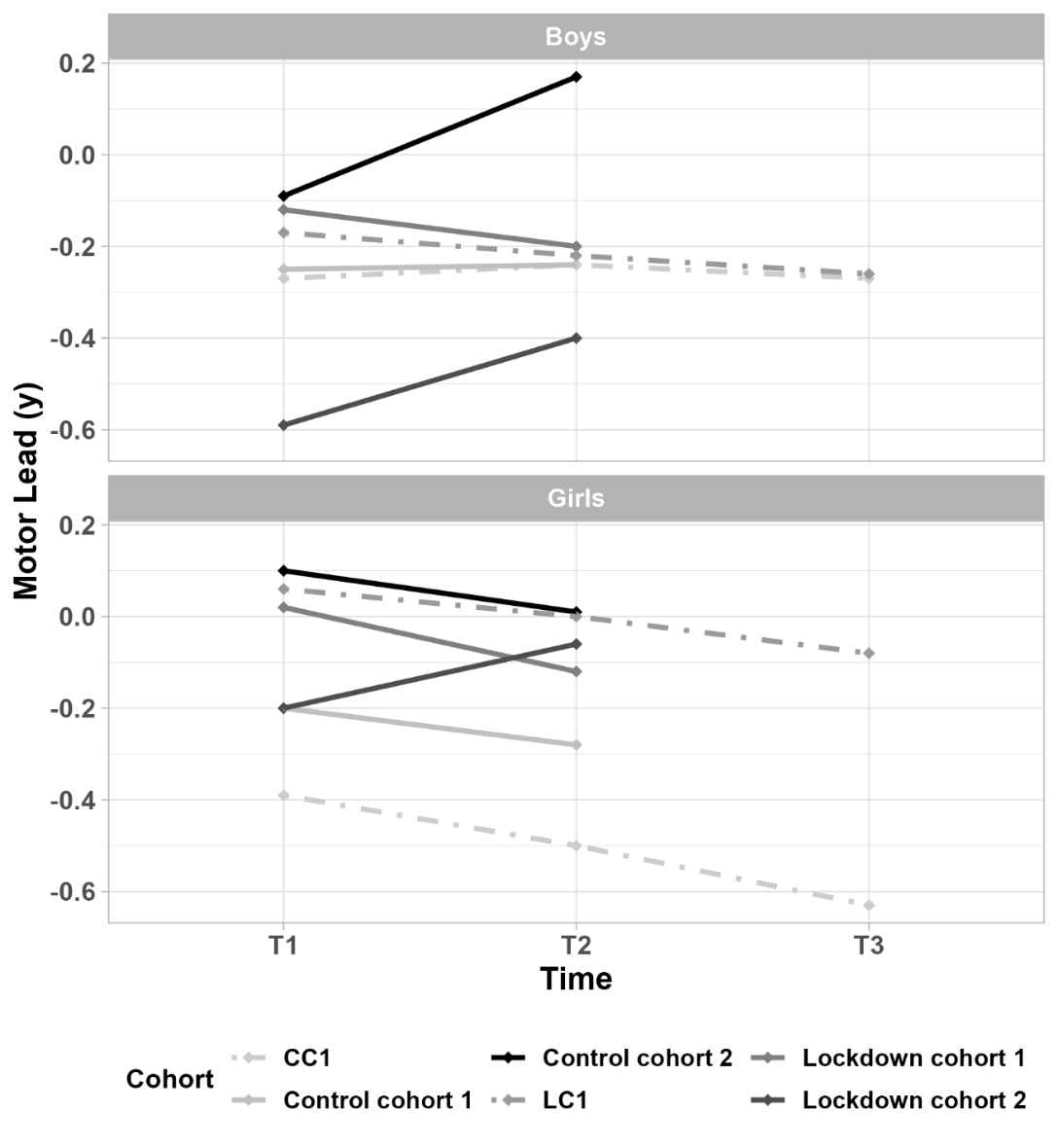


**Appendix Figure 1.** The development of motor lead in years in boys and girls from T1 to T2 for the four different cohorts and from T1-T2-T3 for the two follow-up cohorts (control cohort 1, lockdown cohort 1). Two-year follow-up is done in only 7 schools, which accounts for the differences observed between the samples.


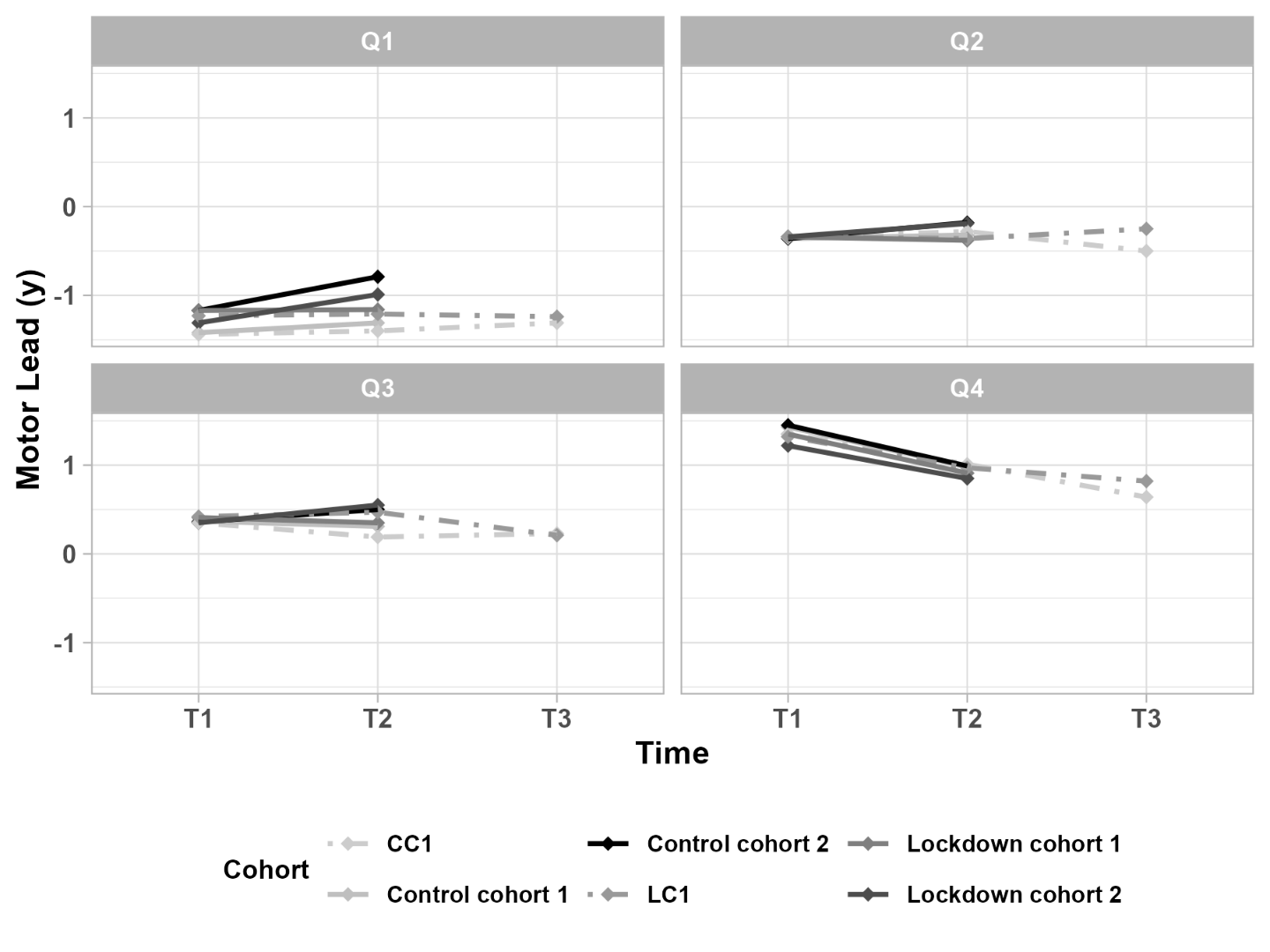


**Appendix Figure 2.** The development of motor lead in years in the four motor ability groups (quartiles) from T1 to T2 for the four different cohorts and from T1-T2-T3 for the two follow-up cohorts (control cohort 1, lockdown cohort 1). Two-year follow-up is done in only 7 schools, which accounts for the differences observed between the samples.

| **Appendix Table 1:** *Multiple Comparisons with Bonferroni correction between the four cohorts on the difference between motor lead on T1 and T2.* | | | | |
| --- | --- | --- | --- | --- |
| Cohort (I) | Cohort (J) | Mean Difference (I-J) | Standard Error | p |
| Control cohort 1 | Control cohort 2 | -,108 | ,077 | ,957 |
| Control cohort 1 | Lockdown cohort 1 | ,066 | ,080 | 1,000 |
| Control cohort 1 | Lockdown cohort 2 | -,203 | ,077 | ,051 |
| Control cohort 2 | Lockdown cohort 1 | ,174 | ,083 | ,224 |
| Control cohort 2 | Lockdown cohort 2 | -,095 | ,081 | 1,000 |
| Lockdown cohort 1 | Lockdown cohort 2 | -,269^*^ | ,084 | ,008* |

## Tables

**** indicates a significant difference on the p < 0.05 level.***

| **Appendix Table 2.** *Analysis on the role of sex in the difference in motor lead (in years) development from T1 and T2 between the 4 cohorts.* | | | | | |
| --- | --- | --- | --- | --- | --- |
|  | Boys | | Girls | |  |
|  | Motor Lead T1 *(M ± SD)* | Motor Lead T2 *(M ± SD)* | Motor Lead T1 *(M ± SD)* | Motor Lead T2 *(M ± SD)* |  |
| **Control cohort 1** | -0,25 ± 1,14 | -0,24 ± 1,26 | -0,20 ± 1,05 | -0,28 ± 1,15 | *Time: F(1,984) = 0,758, p = 0,384*  *Time*cohort: F(3, 984) = 4,347,*  *p = 0,005*  *Time*sexe: F(1,984) = 5,413,*  *p = 0,020 Time*cohort*sexe: F(3,984) = 1.568, p = 0,196*. |
| **Control cohort 2** | -0,09 ± 1,08 | 0,17 ± 1,22 | 0,10 ± 1,04 | 0,01 ± 1,13 |  |
| **Lockdown cohort 1** | -0,12 ± 1,00 | -0,20 ± 1,33 | 0,02 ± 0,86 | -0,12 ± 1,04 |  |
| **Lockdown cohort 2** | -0,59 ± 0,96 | -0,40 ± 1,20 | -0,20 ± 0,88 | -0,06 ± 1,02 |  |

| **Appendix Table 3.** *Analysis on the role of SES in the difference in motor lead (in years) development from T1 and T2 between the 4 cohorts.* | | | | |
| --- | --- | --- | --- | --- |
|  | Motor Lead difference T2 – T1 *(M ± SD)* | | |  |
|  | Low SES  (N = 441) | Medium SES  (N = 375) | High SES  (N = 176) |  |
| **Control cohort 1** | -0,06 ± 0,87^****^ | 0,00 ± 0,81 | -0,11 ± 0,93^**^ | *Time: F(1,980) = 0.021,  p = 0.884. Time*cohort: F(3,980) = 5.634,  p < 0.001. Time*SES: F(2,980) = 0.836,  p = 0.434. Time*cohort*SES:   F(6,980) = 4.800, p < 0,001.* |
| **Control cohort 2** | -0,10 ± 0,99^****^ | 0,10 ± 0,99 | 0,36 ± 0,90^*,*** ^^ |  |
| **Lockdown cohort 1** | -0,12 ± 0,87^****^ | 0,11 ± 0,88 | -0,53 ± 0,73^**,**** ^^ |  |
| **Lockdown cohort 2** | 0,29 ± 0,90^*,**, *** ^^ | 0,01 ± 0,74 | 0,09 ± 0,79^***^ |  |
|  | *Time = F(1,437) = 0,007, p = 0,934. Time*cohort =   F(3,437) = 5,149,  p = 0,002.* | *Time = F(1,371) = 1,530, p = 0,217. Time*cohort =   F(3,371) = 0,401,  p = 0,752.* | *Time = F(1,172) = 0,500, p = 0,480. Time*cohort =   F(3,172) = 8,589,  p < 0,001.* |  |

^*^different from control cohort 1, ^**^ different from control cohort 2, ^***^ different from lockdown cohort 1, ^****^ different from lockdown cohort 2. ^significant change in motor lead from T1 to T2.

| **Appendix Table 4.** *Analysis on the role of motor ability in the difference in motor lead (in years) development from T1 to T2 between the 4 cohorts.* | | | | | |
| --- | --- | --- | --- | --- | --- |
|  | Q1 (N = 310) | | Q2 (N = 280) | |  |
|  | T1 (M ± SD) | T2 (M ± SD) | T1 (M ± SD) | T2 (M ± SD) |  |
| **Control cohort 1** | -1,42 ± 0,62 | -1,31 ± 0,95 | -0,36 ± -0,21 | -0,32 ± 0,86 | *Time: F(1,976)=*  *0.571, p = 0,450*  *Time*cohort: F(3,976)= 3.204, p = 0.023.*  *Time*motor ability: F(3,976) = 20,267,*  *p < 0.001.*  *Time*cohort*motor ability:*  *F(9,976) = 0.420 ,*  *p = 0.925).* |
| **Control cohort 2** | -1,17 ± 0,36 | -0,79 ± 0,95 | -0,36 ± 0,19 | -0,18 ± 0,80 |  |
| **Lockdown cohort 1** | -1,17± 0,44 | -1,16 ± 0,98 | -0,34 ± 0,20 | -0,38 ± 0,84 |  |
| **Lockdown cohort 2** | -1,31 ± 0,45 | -0,99 ± 0,95 | -0,34 ± 0,19 | -0,19 ± 0,83 |  |
|  | Q3  (N = 222) | | Q4  (N = 180) | |  |
|  | T1 (M ± SD) | T2 (M ± SD) | T1 (M ± SD) | T2 (M ± SD) |  |
| **Control cohort 1** | 0,37 ± 0,23 | 0,31 ± 0,81 | 1,43 ± 0,53 | 0,98 ± 0,82 |  |
| **Control cohort 2** | 0,36 ± 0,22 | 0,50 ± 0,85 | 1,45 ± 0,52 | 0,99 ± 1,14 |  |
| **Lockdown cohort 1** | 0,41 ± 0,22 | 0,35 ± 0,98 | 1,35 ± 0,52 | 0,91 ± 1,06 |  |
| **Lockdown cohort 2** | 0,35 ± 0,21 | 0,55 ± 0,85 | 1,22 ± 0,42 | 0,85 ± 0,75 |  |

| **Appendix Table 6.** *Analysis on the role of sex in the difference in motor lead (in years) development from T1 to T3 between the 2 cohorts.* | | | | | | |
| --- | --- | --- | --- | --- | --- | --- |
|  |  | Sample size N | T1  (M *± SD* | T2 (M *± SD* | T3  (M *± SD)* |  |
| **Boys** | **Control cohort 1** | 76 | -0,27 *±* 1,08 | -0.24 *±* 1.22 | -0.27 *±* 1.26 | *Time: F(2,596) = 2.449, p = 0.087*  *Time*cohort: F(2,596) = 0.024, p = 0.976*  *Time*sexe: F(2,596) = 0.927, p = 0.396 Time*cohort*sexe: F(2,596) = 0.408, p = 0,665* |
|  | **Lockdown cohort 1** | 67 | -0,17 ± 0,97 | -0,22 *±* 1,40 | -0,26 *±* 1,28 |  |
| **Girls** | **Control cohort 1** | 90 | -0,39 ± 1,07 | -0.50 *±* 1.24 | -0.63 *±* 1.18 |  |
|  | **Lockdown cohort 1** | 69 | 0,06 ± 0,87 | 0,00 *±* 1,08 | -0,08 *±* 1,09 |  |

| **Appendix Table 5.** *Means and standard deviations of motor lead (in years) for T1, T2 and T3 in the two cohorts* | | | | | | | | |
| --- | --- | --- | --- | --- | --- | --- | --- | --- |
| **Cohort** | **Sample size (N)** | **Sexe (n ,  % boys - girls)** | **Age (y) T1 (M ± SD)** | **Motor Lead T1 (M ± SD)** | **Motor Lead T2 (M ± SD)** | **Motor Lead T3 (M ± SD)** | **t (Paired samples)** | **F** |
| **Control cohort 1** | 166 | 76 – 90  (45.8 – 54.2%) | 6.58 ± 0.44 | -0,34 *±* 1,07 | -0,38 *±* 1,23 | -0,47 *±* 1,22 | *T1-T2: t(165) = 0.707, p = 0.480 T2-T3: t(165) = 1.241, p = 0.217*  *T1-T3: t(165) = 1.747, p = 0.083* | *Time:*  *F(2,600) = 2.681, p = 0.069)*  *Time*Cohort:*  *F(2,600) = 0.033, p = 0.968)* |
| **Lockdown cohort 1** | 136 | 67 –69  (49.3 – 50.7%) | 6.46 ± 0.41 | -0,05 *±* 0,93 | -0,11 *±* 1,25 | -0,17 *±* 1,19 | *T1-T2: t(135) = 0.731, p = 0.466*  *T2-T3: t(135) =-0.675, p = 0.501*  *T1-T3: t(135) = 1.417, p = 0.159* |  |

| **Appendix Table 8.**  *Analysis on the role of SES in the difference in motor lead (in years) development from T1 to T3 between the 2 cohorts.* | | | | | | | |
| --- | --- | --- | --- | --- | --- | --- | --- |
|  |  | Sample size (N) | T1  (M ± SD) | T2 (M ± SD) | T3  (M ± SD) | T  (Paired samples) |  |
| Low SES | **Control cohort 1** | 89 | -0,27 ± 1,20 | -0,37 *±* 1,31 | -0,52 *±* 1,31 | *T1-T2: t(88) = 1.137 , p = 0.259 T2-T3: t(88) = 1.540, p = 0.127*  *T1-T3: t(88) = 2.143, p = 0.035** | *Time: F(2,596) = 3.218, p = 0,041**  *Time*cohort: F(2,596) = 0.037, p = 0.964*  *Time*SES: F(2,596) = 1.365, p = 0.256*  *Time*cohort*SES:  F(2,596) = 4.462,  p = .0.012** |
|  | **Lockdown cohort 1** | 83 | -0,02 ± 0,97 | -0,15 *±* 1,24 | -0,04 *±* 1,20 | *T1-T2: t(82) =1.383, p = 1.71 T2-T3: t(82) =*  *-1.022, p = 0.310*  *T1-T3: t(82) =0.124, p = 0.902* |  |
|  |  |  | *^Time: F(1,898, 322.61) = 1,928, p = 0,149*  *^Time*cohort: F(1.898,* 322.61*) = 2.019, p = 0.137* | | | |  |
| Medium SES | **Control cohort 1** | 77 | -0,41 ± 0,91 | -0,40 *±* 1,14 | -0,41 *±* 1,12 | *T1-T2: t(76) =*  *-0.167 , p = 0.868 T2-T3: t(76) = 0.109, p = 0.914*  *T1-T3: t(76) = -0.064, p = 0.949* |  |
|  | **Lockdown cohort 1** | 53 | -0,10 ± 0,85 | -0,04 *±* 1,26 | -0,37 *±* 1,15 | *T1-T2: t(52) =*  *-0.455 , p = 0.651 T2-T3: t(52) = 2.723 , p = 0.009**  *T1-T3: t(52) = 2.172, p = 0.034** |  |
|  |  |  | *Time: F(*2*,256) = 2.744, p = 0*,066  *Time*cohort: F(2,256) = 2.580, p = 0.078* | | | |  |

| **Appendix Table 7.** *Analysis on the role of motor ability in the difference in motor lead (in years) development from T1 to T3 between the 2 cohorts.* | | | | | | |
| --- | --- | --- | --- | --- | --- | --- |
|  |  | Sample size (N) | T1  (M *±* SD) | T2 (M *±* SD) | T3  (M *±* SD) |  |
| **Q1** | **Control cohort 1** | 57 | -1,44 ± 0,70 | -1,40 ± 0,95 | -1,31 ± 1,03 | *Time:*  *F(2, 588) = 5.757,*  *p = 0.003* Time*cohort:*  *F(2, 588) = 0.131,*  *p = 0.877*  *Time*motor ability: F(6,588) = 3.017,*  *p = 0.007* Time*cohort*motor ability:  F(6,588)) = 1.088,  p = 0.368* |
|  | **Lockdown cohort 1** | 31 | -1,23 ± 0,50 | -1,21 ± 1,06 | -1,24 ± 0,99 |  |
| **Q2** | **Control cohort 1** | 51 | -0,35 ± 0,22 | -0,28 ± 0,96 | -0,50 ± 0,96 |  |
|  | **Lockdown cohort 1** | 47 | -0,34 ± 0,21 | -0,36 ± 0,82 | -0,25 ± 1,04 |  |
| **Q3** | **Control cohort 1** | 34 | 0,35 ± 0,22 | 0,19 ± 0,81 | 0,23 ± 1,02 |  |
|  | **Lockdown cohort 1** | 33 | 0,42 ± 0,23 | 0,47 ± 1,03 | 0,21 ± 0,85 |  |
| **Q4** | **Control cohort 1** | 24 | 1,35 ± 0,50 | 1,01 ± 0,83 | 0,64 ± 0,95 |  |
|  | **Lockdown cohort 1** | 25 | 1,32 ± 0,39 | 0,97 ± 1,13 | 0,82 ± 0,97 |  |

** indicates a significant difference or effect at the p < 0.05 level.
^The assumption of sphericity was violated χ^2^(2) = 9.364, p = 0.009, therefore the Greenhouse-Geisser correction was used.
* indicates a significant difference or effect at the p < 0.05 level.*
